# A Systemic Review of Adolescent Supracondylar Fractures: What Is the Surgical Treatment, Open Reduction With Internal Fixation (ORIF) or Closed Reduction With Percutaneous Pinning (CRPP)?

**DOI:** 10.7759/cureus.58123

**Published:** 2024-04-12

**Authors:** Shahad O Alshaynawi, Abdullah A Alshomrany, Abdullah Alshebromi, Amal Alsulami, Fatimah H Aleid, Hanan Al Kaabi, Khalid F Alrasheed, Razan Alotaibi, Eyad A Alakkas

**Affiliations:** 1 Surgery, King Abdulaziz University Hospital, Jeddah, SAU; 2 College of Medicine, University of Bisha, Bisha, SAU; 3 Orthopedic Surgery, King Fahad Specialist Hospital, Qassim, SAU; 4 Medicine and Surgery, King Abdulaziz University Faculty of Medicine, Jeddah, SAU; 5 Orthopedic Surgery, Alfaisal University College of Medicine, Riyadh, SAU; 6 Surgery, College of Medicine, Abha, SAU; 7 Medical Student, King Saud University, Riyadh, SAU; 8 Orthopedic Surgery, King Saud Medical City, Riyadh, SAU; 9 Orthopedic Surgery, King Faisal Specialist Hospital & Research Centre, Jeddah, SAU

**Keywords:** supracondylar humeral fracture, closed reduction with percutaneous pinning, open reduction with internal fixation, adolescents, a systematic review

## Abstract

Supracondylar humerus (SCH) fractures represent the most frequent elbow injury in young children. These fractures can be treated through either closed reduction with percutaneous pinning (CRPP) or open reduction with internal fixation (ORIF). Yet, the optimal treatment option for adolescents remains unclear. This research contrasts the results of CRPP and ORIF treatments for distal humerus fractures in adolescents.

In June 2023, we conducted a comprehensive search of PUBMED, OVID MEDLINE, Web of Science, Cochrane Central Register of Controlled Trials, and various trial registries without any time restrictions. We evaluated the quality of qualifying studies using the Methodological Index for Non-randomized Studies (MINORS) and Cochrane risk measures for bias. We extracted data particularly related to patient demographics, fracture details, medical procedures followed, complications encountered, and the resulting outcomes.

Out of the 488 studies identified, only four satisfied the inclusion criteria. Both methods illustrated comparable outcomes in terms of range of motion, averaging approximately 118 degrees in the ORIF group versus a span of 114 to 128 degrees in the CRPP group. The immobilization period varied, spanning 10 to 13 days for ORIF versus 24 to 29 days for CRPP. Despite this, CRPP displayed a decreased necessity for additional surgery. Notably, one study indicated a higher frequency of heterotopic ossification within the ORIF group.

This review indicates that both CRPP and ORIF are effective for treating supracondylar fractures in adolescents, yielding similar results. However, CRPP has a lower need for follow-up surgery. Future studies with larger sample sizes are needed to solidify these findings, providing stronger guidance for treatment.

## Introduction and background

Supracondylar humerus (SCH) fractures are the most common elbow injury in children under 10 years old [1], with reported instances ranging from 3.3% to 16.6% [2]. Their frequency decreases as children mature. Recent studies found no significant differences between the outcomes of closed reduction percutaneous pinning (CRPP) and open reduction internal fixation (ORIF) in this demographic, with infection being the primary postoperation complication in both methods [3,4]. Neither method demonstrated a significant advantage over the other [3]. However, studies showed significantly better outcomes for children with Gartland type 3 SCH fractures treated with ORIF, reinforcing ORIF’s superiority over CRPP, albeit these studies had limited sample sizes [5,6].

In contrast, a review of patients aged 10 to 17 years showed CRPP was a preferable treatment for extra-articular fractures, leading to predictable outcomes and fewer complications than ORIF [1], which was typically required for intra-articular fractures. However, the success recorded for surgical treatment of the latter was not consistently excellent, often producing high complications [7].

CRPP proves reliable for adolescents with extra-articular supra-condylar fractures and is typically followed by three to four weeks of immobilization for children with SCH fractures [8,9]. For adults, ORIF is the standard, as data suggest that long-term elbow immobilization negatively affects motion recovery. Nonetheless, there’s a lack of consensus on the most effective treatment method for adolescents. Therefore, our goal is to assess and compare the outcomes of CRPP and ORIF for distal humerus fractures in adolescents.

## Review

Materials and methods

This systematic review adhered to the guidelines of the Preferred Reporting Items for Systematic Reviews and Meta-Analyses (PRISMA) [10]. The study protocol was preemptively registered in the National Institute of Health Research’s Prospective Register of Systematic Reviews, PROSPERO, under registration number CRD42023442467 [11].

Search Strategies

The following databases were searched in June 2023: PUBMED, OVID MEDLINE, Web of Science, the Cochrane Central Register of Controlled Trials (CENTRAL), and trial registries (ClinicalTrials.gov, MetaRegister of Controlled Trials, Australian New Zealand Clinical Trials Registry, UMIN Clinical Trials Registry). Both text words and medical subject heading (MeSH) terms (all fields) were used as follows: (Distal humerus fracture OR Supracondylar Humeral Fracture) AND (open reduction OR internal fixation OR ORIF) AND (closed reduction OR percutaneous pinning OR CRPP) AND (Adolescents OR Older children). Each reference in the articles that were included was additionally evaluated for relevance. The most recent or comprehensive report was utilized, where there were several published reports describing the same sample.

Eligibility Criteria

This review encompasses all cohort studies, randomized controlled trials (RCTs), and non-RCTRs published in English without time frame restrictions that report the number of adolescent patients (10-16 years old) undergoing CRPP and ORIF. Any study failing to meet these inclusion criteria was omitted. This exclusion encompasses studies published in non-English languages, those that include pediatric or adult patients, those not using CRPP or ORIF as a treatment for distal humerus fracture, those including isolated fractures of the medial epicondyle or lateral condyle or ipsilateral limb fractures, and studies assessed as exhibiting a high bias risk or low quality based on an analysis of study design, sample size, data collection and analysis, and other pertinent factors.

Screening and Selection of Studies

After performing a database search, Rayyan Software processed all the collected data. Each of the four authors independently examined the titles and abstracts of every study identified in the search to pinpoint potentially relevant papers. Full-text articles were acquired for studies that met the inclusion criteria, and the authors independently evaluated them for suitability. In the event of a disagreement, the senior author was sought for resolution. The screening process was documented using a PRISMA flow diagram, including the factors leading to exclusion.

Data Extraction

The four authors diligently extracted data from the text, tables, and figures of the studies included, utilizing a standardized form designed in Microsoft Excel. They saved the completed data extraction form securely on a password-protected laptop and in Google Drive. They extracted the following data: the first author, publication details (journal name, country of origin, year of publication), study design, sample size; participant information (age, gender, weight, co-morbidities); injury details (mechanism, open or closed fracture status, neurological and vascular status); treatment details (type of reduction, reported complications, revision rate, additional treatments, overall results); 1-year follow-up outcomes, and any clinical recommendations.

Risk of Bias and Quality Assessment

Two reviewers independently used the Methodological Index for Non-randomized Studies (MINORS) to assess the potential bias in both retrospective and prospective non-randomized studies [12]. This validated 12-item tool is designed to evaluate the integrity of non-randomized surgical studies. Simultaneously, the quality of RCTs was assessed by two independent authors using the Cochrane risk tool for bias [13]. Each tool was combined to give an overall risk of bias 2 (RoB2) judgment across five major domains, adhering to established standards. Our focus was on trial design, execution, and reporting while utilizing a series of “signaling questions” to extract pertinent information regarding the risk of bias. This was then evaluated using an algorithm, and the findings were categorized as either “low” (all domains presenting low bias), “some concerns” (at least one domain presenting some level of concern), or “high” (one or more domains presenting high risk or multiple domains raising some concerns). In cases where information was missing from an article, the authors were contacted to provide the necessary data. Any missing content and its potential effects on the review’s outcomes were thoroughly explained if said data could not be procured.

Results

Search Results

**Figure 1:**
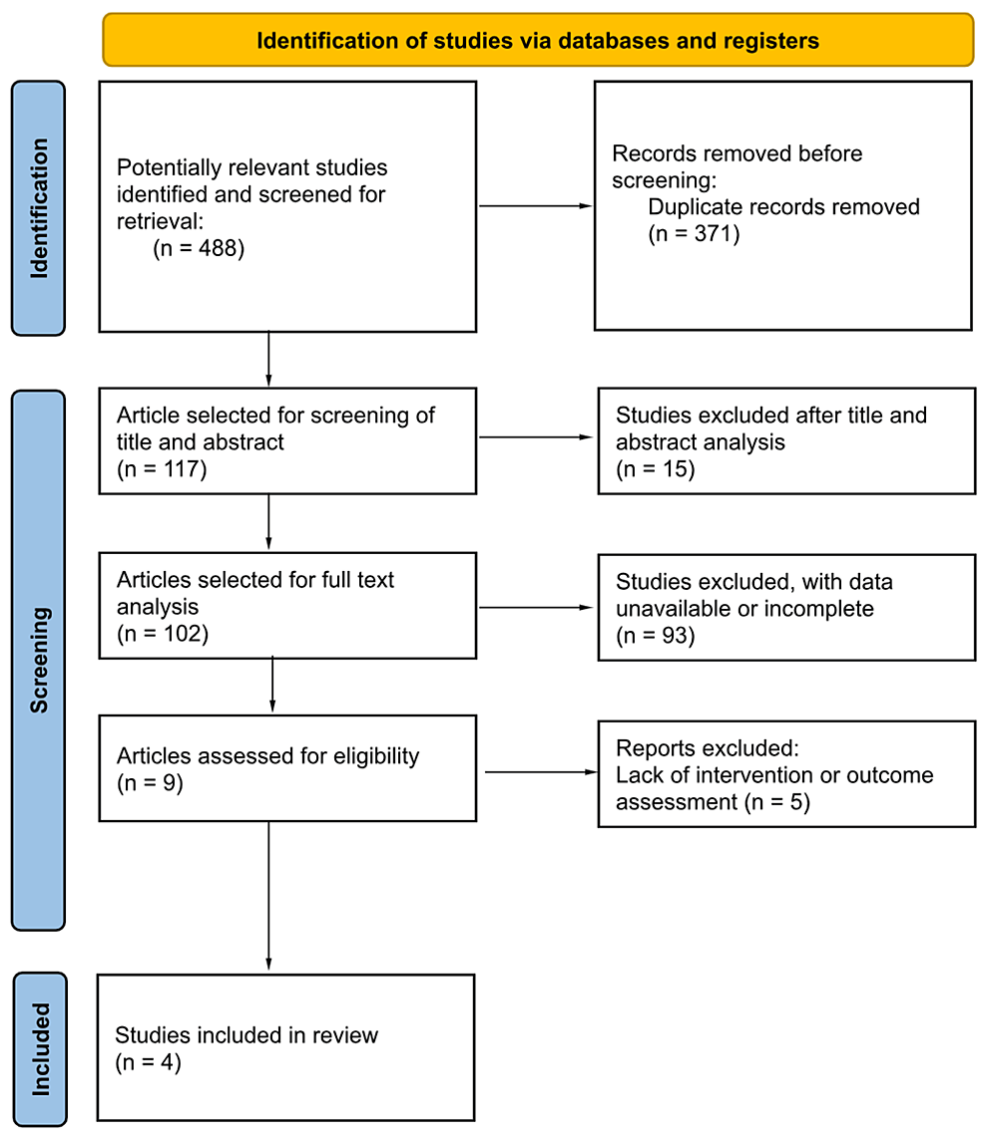
PRISMA flowchart depicting the selection of articles PRISMA: Preferred Reporting Items for Systematic Reviews and Meta-Analyses

Figure 1 illustrates the data selection process. An initial search of the computerized database yielded 488 documents. Duplication led to the removal of 371 articles before screening. Of the 117 articles screened based on title and abstract, 15 were excluded. A total of 102 articles were then selected for full-text analysis. Among these, 93 were discarded due to unavailable or incomplete data. Following evaluation for intervention and outcome, nine articles were deemed suitable, but five were subsequently excluded for irrelevance. Consequently, four relevant articles were included in the current review.

Study Characteristics

This review incorporates four eligible studies, each subjected to an independent double review. The articles were published from 2013 (Julfiqar) to 2020 (Allah Rakha), with the majority released in 2020 [14,15]. Four of the studies included 143 cases in total: 93 cases in the ORIF group and 50 in the CRPP group.

The typical patient age ranged from approximately 11 to 16 years in Julfiqar’s CRPP study [15]. Predominantly, the patients were male, with females representing less than half in all studies. None of the cases reported any co-morbidities. The primary source of injuries was falls, although there were some instances of road traffic accidents (RTAs) and blunt trauma injuries.

For injury classification, two studies from Allah Rakha and Mingjing Li stated that patients had Gartland type III injuries [14,16]. Three studies reported cases of extra-articular fractures, and just one cited intra-articular fractures. Closed fractures were reported in two studies while the others recorded both open and closed fractures. Nerve deficits were documented in three studies while others indicated no neurological issues.

In terms of treatment, three studies performed CRPP and ORIF procedures while one study focused solely on ORIF. Concerning clinical outcomes, a single study reported heterotopic ossification in 33% of ORIF and 3% of CRPP patients. No instances of revision surgeries were noted in the studies.

The average range of movement for the ORIF group was approximately 118 degrees compared to a range of 114 to 128 degrees for the CRPP group. The immobilization duration varied from 10 to 13 days in the ORIF cases to 24 to 29 days in the CRPP cases. Subsequent surgeries varied from 27.6% to 50% in ORIF cases and from 0% to 12.5% in the CRPP group. The average time to achieve maximum motion was about 3.8 months for the ORIF group and 3.0 months for the CRPP group. According to one study, both ORIF and CRPP treatments were equally effective, as indicated by Flynn’s criteria (Table 1).

**Table 1:** Study characteristics ORIF: open reduction internal fixation; CRPP: closed reduction percutaneous pinning

References	Country	Study design	Total number of patients in each group		Age		Sex (Male)		Outcomes		Type of reduction (CRPP or ORIF)
			ORIF	CRPP	ORIF	CRPP	ORIF	CRPP	ORIF	CRPP	
Bell et al. [1]	United States	Retrospective review	6	38	11.3± 1.4	11.4± 1.4	4 (67%)	29 (82%)	Range of motion (degrees.) 118± 13.	Range of motion (degrees) 128± 10.	Both
Allah Rakha et al. [14]	Pakistan	Randomized clinical trial.	4	5	11 - 12	11 - 12	-	-	-	-	Both
Mingjing Li et al. [16]	China	Retrospective review	83	0	more than 10	0	61 (73.5%)		Using Flynn’s criteria showed that the efficacy of ORIF and CRPP were the same.		ORIF
Julfiqar et al. [15]	India	Retrospective review	0	7	0	12-16	0	6	-	The average Mayo Elbow Performance Score (MEPS) for six patients was 85, which was rated as good.	CRPP

Discussion

The occurrence of supracondylar humeral (SCH) fractures tends to lessen as children grow older and near skeletal maturity [1,17]. This is because bone mass usually increases during the transition into adolescence, which can, in turn, affect the pattern of fractures and their respective treatment plans [17]. SCH fractures are categorized under Gartland’s criteria as either non-displaced fractures (type I), displaced fractures with an intact posterior cortex (type II), or completely displaced fractures (type III) [18]. Extension-type fractures are the most common form of SCH fractures. Conversely, flexion-type fractures are rare, making up only 1-10% of all SCH fractures [17].

Nonetheless, the incidence of flexion-type SCH fractures appears to be higher in adolescents [17]. To our knowledge, this is the first systematic review to report on the outcomes of surgical treatments for adolescents with SCH fractures. As such, our objective is to evaluate and compare the results of CRPP and ORIF in managing distal humerus fractures in adolescents.

Phillip et al. highlighted a decreasing trend in extra-articular injuries coupled with an increase in intra-articular injuries as patients neared skeletal maturity [1]. Most outcome parameters, such as range of motion, immobilization duration, need for a second surgery, and average time to maximum motion, were similar between the two procedures. However, the need for secondary surgery was lower in the CRPP group while heterotopic ossification was higher in the ORIF group [1]. Hence, it is recommended to use CRPP for displaced supracondylar humerus fractures in children [19]. Despite this, both ORIF and CRPP methods show equivalent efficacy in SCH fracture treatment according to Flynn’s criteria [14].

Julfiqar et al. conducted CRPP for an adolescent with intercondylar fracture, which revealed a pin tract infection treated conservatively and mild elbow stiffness [15] as compared to ORIF, as it raises the chance of elbow stiffness and causes additional damage to the surrounding soft tissues [20-22], with a noted nerve deficit developing in most cases including ORIF [16,22].

In conclusion, the choice between CRPP and ORIF for treating supracondylar fractures in adolescents presents a challenge for surgeons. Existing data fails to clearly favor one method over the other. Furthermore, it remains unclear whether adolescents should be treated as children or adults, despite our review of studies comparing CRPP and ORIF outcomes in children and showing no significant difference between the two approaches.

This systematic review has a few limitations. First, it only includes four studies and a single randomized clinical trial, which restricts the breadth of data. Lastly, the variability in internal fixation methods could lead to inconsistent outcomes.

## Conclusions

In summary, the studies we reviewed show that both ORIF and CRPP are effective in treating supracondylar fractures in adolescents, achieving similar results. However, fewer subsequent surgeries are required with CRPP. It is important to acknowledge the limitations of our systematic review, namely, the select nature of the studies included and the relatively small sample size. Future research comparing the two surgical options should involve a larger sample size.

## References

[1] Bell P, Scannell BP, Loeffler BJ (2017). Adolescent distal humerus fractures: ORIF versus CRPP. J Pediatr Orthop.

[2] Shenoy PM, Islam A, Puri R (2020). Current management of paediatric supracondylar fractures of the humerus. Cureus.

[3] Zhu S, Zheng Y, Jiang Y, Yin H, Zhu D (2023). Open versus closed reduction internal fixation for lateral condyle humeral fractures in children: a systematic review and meta-analysis. J Orthop Surg Res.

[4] Astawa P, Maharjana MA, Adisthanaya S, Satya Putra MW, Suarjaya Putra A, Savio SD (2021). Close reduction percutaneous pinning (CRPP) versus open reduction internal fixation (ORIF) for pediatric supracondylar humerus fractures Gartland type II and III: a systematic review and meta-analysis. J Indon Med Assoc.

[5] Abousaleh MA, Zeidan AA, Mukhtar I (2022). Comparative effectiveness of closed reduction with percutaneous pinning and open reduction with internal fixation in the operative management of pediatric type III supracondylar fractures. Cureus.

[6] Ali P, Khan KM, Khoso RE, Soomro S, Kumar R (2023). Comparison of the functional outcomes of close reduction percutaneous pinning versus open reduction internal fixation with pinning in children with Gartland type III supracondylar fracture of humerus. Pak Armed Forces Med J.

[7] Cook JB, Riccio AI, Anderson T, Chen W, Shaha SH, Wimberly RL (2016). Outcomes after surgical treatment of adolescent intra-articular distal humerus fractures. J Pediatr Orthop.

[8] Bekmez S, Camp MW, Ling R, El-Amiri N, Howard AW (2021). Supracondylar humerus fractures in older children: success of closed reduction and percutaneous pinning. J Pediatr Orthop.

[9] Waddell JP, Hatch J, Richards R (1988). Supracondylar fractures of the humerus--results of surgical treatment. J Trauma.

[10] Page MJ, McKenzie JE, Bossuyt PM (2021). The PRISMA 2020 statement: an updated guideline for reporting systematic reviews. BMJ.

[11] Schiavo JH (2019). PROSPERO: an international register of systematic review protocols. Med Ref Serv Q.

[12] Slim K, Nini E, Forestier D, Kwiatkowski F, Panis Y, Chipponi J (2003). Methodological index for non-randomized studies (minors): development and validation of a new instrument. ANZ J Surg.

[13] Sterne JA, Savović J, Page MJ (2019). RoB 2: a revised tool for assessing risk of bias in randomised trials. BMJ.

[14] Rakha A, Khan RDA, Arshad A, Khan ZA, Ahmad S, Mahmood S (2020). Comparison of efficacy between open and close reduction in supracondylar fracture of humerus in children using Flynn’s criteria. Ann Punjab Med Coll.

[15] Julfiqar Julfiqar, Pant A, Huda N, Ahmed W (2013). Closed reductions and percutaneus 'k' wire fixation for adolescent intercondylar fractures of the distal humerus. J Clin Diagn Res.

[16] Li M, Xu J, Hu T, Zhang M, Li F (2019). Surgical management of Gartland type III supracondylar humerus fractures in older children: a retrospective study. J Pediatr Orthop B.

[17] Yangs CP (2020). Fracture patterns and surgical outcomes of supracondylar humeral fractures in adolescents. Formosan Journal of Musculoskeletal Disorders.

[18] GA JJ (1959). Management of supracondylar fractures of the humerus in children. Surg Gynecol Obstet.

[19] Brighton B, Abzug J, Ho CA, Ritzman TF (2016). Current strategies for the management of pediatric supracondylar humerus fractures: tips and techniques for successful closed treatment. Instr Course Lect.

[20] Gupta R (1996). Intercondylar fractures of the distal humerus in adults. Injury.

[21] Kundel K, Braun W, Wieberneit J, Rüter A (1996). Intraarticular distal humerus fractures. Factors affecting functional outcome. Clin Orthop.

[22] Mok CY, Lui TH (2013). T-condylar fractures of the distal humerus in children: report on three cases. BMJ Case Rep.

